# Retraction: Synthesis, characterization and cytotoxicity evaluation of a novel magnetic nanocomposite with iron oxide deposited on cellulose nanofibers with nickel (Fe_3_O_4_@NFC@ONSM-Ni)

**DOI:** 10.1039/d3ra90131a

**Published:** 2024-01-05

**Authors:** Pouya Ghamari kargar, Maryam Noorian, Elham Chamani, Ghodsieh Bagherzade, Zahra Kiani

**Affiliations:** a Department of Chemistry, Faculty of Sciences, University of Birjand Birjand Iran P.ghamari71@gmail.com +98 56 32345192 +98 56 32345192; b Student Research Committee, Birjand University of Medical Sciences Birjand Iran; c Department of Clinical Biochemistry, Birjand University of Medical Sciences Birjand Iran; d Department of Pharmacology, Birjand University of Medical Sciences Birjand Iran Kiani.za@gmail.com gbagherzade@gmail.com bagherzade@birjand.ac.ir +98 56 32381920

## Abstract

Retraction of ‘Synthesis, characterization and cytotoxicity evaluation of a novel magnetic nanocomposite with iron oxide deposited on cellulose nanofibers with nickel (Fe_3_O_4_@NFC@ONSM-Ni)’ by Pouya Ghamari kargar *et al.*, *RSC Adv.*, 2021, **11**, 17413–17430, https://doi.org/10.1039/D1RA01256H.

The Royal Society of Chemistry, with the agreement of the authors, hereby wholly retracts this *RSC Advances* article due to concerns with the reliability of the data.

The XRD patterns in Fig. 4a and c, and the EDX data in Fig. 3a contain repeating sections. The authors provided the raw XRD data for Fig. 4a and c, and raw EDX data for Fig. 3a, but these were found to contain duplicated sections of data across different datasets representing different samples within this article and other articles.

The raw XRD data provided for Fe_3_O_4_ in Fig. 4a of this article was identical in a number of different regions to raw XRD data provided by the authors for CuO in Fig. 4b of ref. 1 and Fig. 3 of ref. 2. In Fig. 4c the raw XRD data for Fe_3_O_4_@NFC@ONSM-Ni was also found to contain identical sections of data within the raw dataset.

The raw EDX data for Fe_3_O_4_ in Fig. 3a was found to contain a number of regions of identical data within the raw dataset. In addition, part of the raw EDX data is identical to regions of raw EDX data provided by the authors for Fe_3_O_4_@NFC-NSaloph(Cu)CO_2_H in Fig. 4c of ref. 2.

The authors have stated that they outsourced the XRD and EDX data collection to an external company.

Given the significance of these concerns, the findings presented in this paper are no longer reliable.

The authors were informed about the retraction of the article. Pouya Ghamari kargar and Ghodsieh Bagherzade have not agreed with the decision. The other authors were contacted but have not responded.

Signed: Laura Fisher, Executive Editor, *RSC Advances*

Date: 13^th^ December 2023

## Supplementary Material
